# GO functional similarity clustering depends on similarity measure, clustering method, and annotation completeness

**DOI:** 10.1186/s12859-019-2752-2

**Published:** 2019-03-27

**Authors:** Meng Liu, Paul D. Thomas

**Affiliations:** Department of Preventive Medicine, Keck School of Medicine, University of Southern California, Los Angeles, USA

**Keywords:** Gene Ontology, semantic similarity, annotation completeness, Directed Acyclic Graphic clustering, hierarchical clustering, least-diverged human orthologs, information content

## Abstract

**Background:**

Biological knowledge, and therefore Gene Ontology annotation sets, for human genes is incomplete. Recent studies have reported that biases in available GO annotations result in biased estimates of functional similarities of genes, but it is still unclear what the effect of incompleteness itself may be, even in the absence of bias. Pairwise gene similarities are used in a number of contexts, including gene “functional similarity” clustering and the related problem of functional ontology structure inference, but it is not known how different similarity measures or clustering methods perform on this task, and how the clusters are affected by annotation completeness.

**Results:**

We developed representations of both “complete” and “incomplete” GO annotation datasets based on experimentally-supported annotations from the GO database—specifically designed to model the incompleteness of human gene annotations—and computed semantic similarities for each set using a variety of different published measures. We then assessed the clusters derived from these measures using two different clustering methods: hierarchical clustering, and the CliXO algorithm. We find the CliXO algorithm, combined with appropriate measures, performs better than hierarchical clustering in reconstructing GO both when the data are complete, and incomplete. Some measures, particularly those that create a pairwise gene similarity by averaging over all pairwise annotation similarities, had consistently poor performance, and a few measures, such as Lin best-matched average and Relevance maximum, were generally among the best performers for a broad range in annotation completeness and types of GO classes. Finally, we show that for semantic similarity-based clustering, the multicellular organism process branch of the GO biological process ontology is more challenging to represent than the cellular process branch.

**Conclusions:**

We assessed the effects of annotation completeness on the distribution of pairwise gene semantic similarity scores, and subsequent effects on the clusters derived from these scores. Our results suggest combinations of semantic similarity measures, gene-level scoring methods and clustering method that perform best for functional gene clustering using annotation sets of varying completeness. Overall, our results underscore the importance of increasing the completeness of GO annotations to for supporting computational analyses of gene function.

**Electronic supplementary material:**

The online version of this article (10.1186/s12859-019-2752-2) contains supplementary material, which is available to authorized users.

## Background

The Gene Ontology (GO), a standardized vocabulary of biological function and process terms, is one of the most frequently used resources for gene function annotations [1]. It consists of 3 domains: molecular function (how a gene functions at the molecular level, e.g. a protein kinase), cellular component (location relative to cell compartments and structures where the gene product is active, e.g. the plasma membrane) and biological process (what larger processes a gene product helps to carry out). Within each domain, the ontology is structured as a directed acyclic graph (DAG) and consists of GO terms that represent different biological properties. Terms low in the DAG are more specific and can have several types of defined relationships to one or more “parent” terms. For the purposes of this paper (grouping genes into biological process classes), we consider two relationship types: “is-a” indicating a child term is a sub-class of its parent term, and “part-of” indicating it is a component of its parent term. It is now common to use the GO in many applications, including gene set enrichment [2–5], gene network [6, 7] and pathway analysis [8, 9].

A GO annotation associates a specific gene (more precisely a gene product, a protein or noncoding RNA, though we use the term “gene” for simplicity) with a specific class (or “term”) in the Gene Ontology, identifying some aspect of its function. Genes annotated to the same molecular function term have a common molecular mechanism of action, e.g. protein kinase activity; genes annotated to the same cellular component term perform their activities in the same cellular compartment or structure; and genes annotated to the same biological process class are involved in the given biological process. All GO annotations also refer to the evidence underlying them, which can be either from a published experiment, or inferred using a computational method. In this paper, we consider only GO annotations supported by experimental evidence.

GO annotations are commonly used in measures that seek to quantify the functional similarity between genes. As each gene is typically annotated with multiple GO terms, functional similarity involves both a measure for the “semantic” similarity between two GO terms, as well as a method for combining multiple pairwise GO term similarities into an overall gene function similarity score. Several proposed GO semantic similarity measures have been published in the literature, and applied in numerous subsequent studies. Most of measures quantify pairwise GO term semantic similarity by the amount of information shared between two terms, i.e. information content (IC) of the most informative (usually also the nearest) common ancestor of two terms. The most highly-cited measures for computing IC-based GO term similarity are Lin’s [10], Jiang and Conrath’s [11], Resnik’s [12] and Schicker’s scores [12]. Overall pairwise *gene level similarities* are computed from the pairwise semantic similarity scores in three distinct ways: 1) using the maximal GO term semantic similarity (MAX), 2) averaging over those best-matched pairwise term semantic similarities (best-match average, or BMA), or 3) averaging over all pairwise term semantic similarities (AVG) [13–15]. In addition to IC-based measures, other measures include graph-based approaches [16] and vector based approaches, e.g. Cosine/vector dot product [17], and Jaccard index [18]. An additional file introduces each similarity measure in more detail [see Additional file 1].

Different studies have evaluated and compared those measures. For example, Resnik’s method has been reported to have the highest correlation with sequence similarity [13, 18], as well as performing best in stratifying protein-protein interactions [19], and the best-match average method of combining GO term semantic similarities was found to perform best overall [18]. More recently, Mazandu and Mulder assessed the performance of different measures in different applications, and found that while BMA approaches (except using Resnik’s measure) correlate best with sequence similarity and functional similarity measures, AVG-based approaches correlate best with protein-protein interaction networks [20].

Pairwise gene semantic similarities are used in a number of contexts, such as for summarizing and visualizing lists of GO terms obtained in enrichment analysis [21], for constructing functional gene modules [22], and perhaps most commonly, for gene ‘functional similarity’ clustering [12, 18]. For functional similarity clustering, most of the published methods create hierarchical clusters. Two types of strategies are generally considered for hierarchical clustering: the agglomerative approach (“bottom up”), and the divisive approach (“top down”). In addition, different linkage criteria are used to determine the distance between objects to be clustered [23, 24]. The major limitation for hierarchical clustering is that it only allows each gene to belong to one cluster. To overcome this limitation, Kramer, et al. developed Clique Extracted Oncology (CliXO) algorithm for Directed Acyclic Graphic (DAG) clustering [25], which allows each gene to belong to different clusters, and for each cluster to have multiple parent clusters. Kramer et al. showed that for at least one similarity measure, CliXO can reconstruct the Gene Ontology (cellular component aspect) to a high degree of accuracy, using the annotations for yeast genes. However, we note that cellular component annotations for yeast genes are relatively complete. It remains unclear how clustering approaches perform in the more common scenario of incomplete annotations. Annotation incompleteness has been shown to be an important confounder in recent efforts to evaluate gene function prediction accuracy [26].

**Fig. 1:**
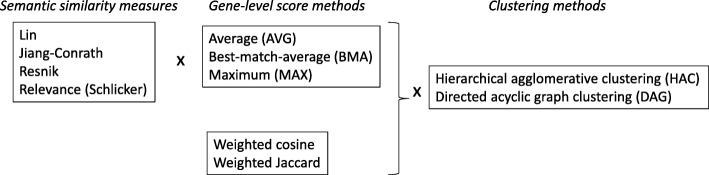
Overall study design. Four different semantic similarity measures were each used to generate gene-level similarities using three different methods, yielding 12 different gene-level measures. Two other measures that are inherently gene level (cosine, Jaccard) were also used, for a total of 14 gene-level measures. Each of these 14 measures were used in two different clustering methods

Here, we evaluate the accuracy and robustness of the most highly used similarity measures to the incompleteness of annotations, focusing on their performance on gene clustering using relevant packages in R [27–29]. We focus on biological process annotations, as these are used for most GO-based functional analyses. First, we create approximations to “completely annotated” *human* gene sets using data from well-studied model organisms, separately for cellular-level, and multicellular organism-level processes. We then roughly quantify the current incompleteness of annotations of human genes. We then use the estimated incompleteness to simulate a large number of incomplete annotation sets. Finally, we evaluate the performance of different similarity measures, and different clustering methods, for both “complete” and “incomplete” annotation sets. The overall study design is shown in Fig. 1. We analyze a total of 14 different gene-level similarity measures (4 different semantic similarity measures × 3 different gene-level scoring methods) + (2 different gene-level measures, cosine and Jaccard), together with two different clustering methods, for a total of 28 unique combinations.

## Results

### Quantifying the incompleteness of knowledge of human gene function

**Fig. 2:**
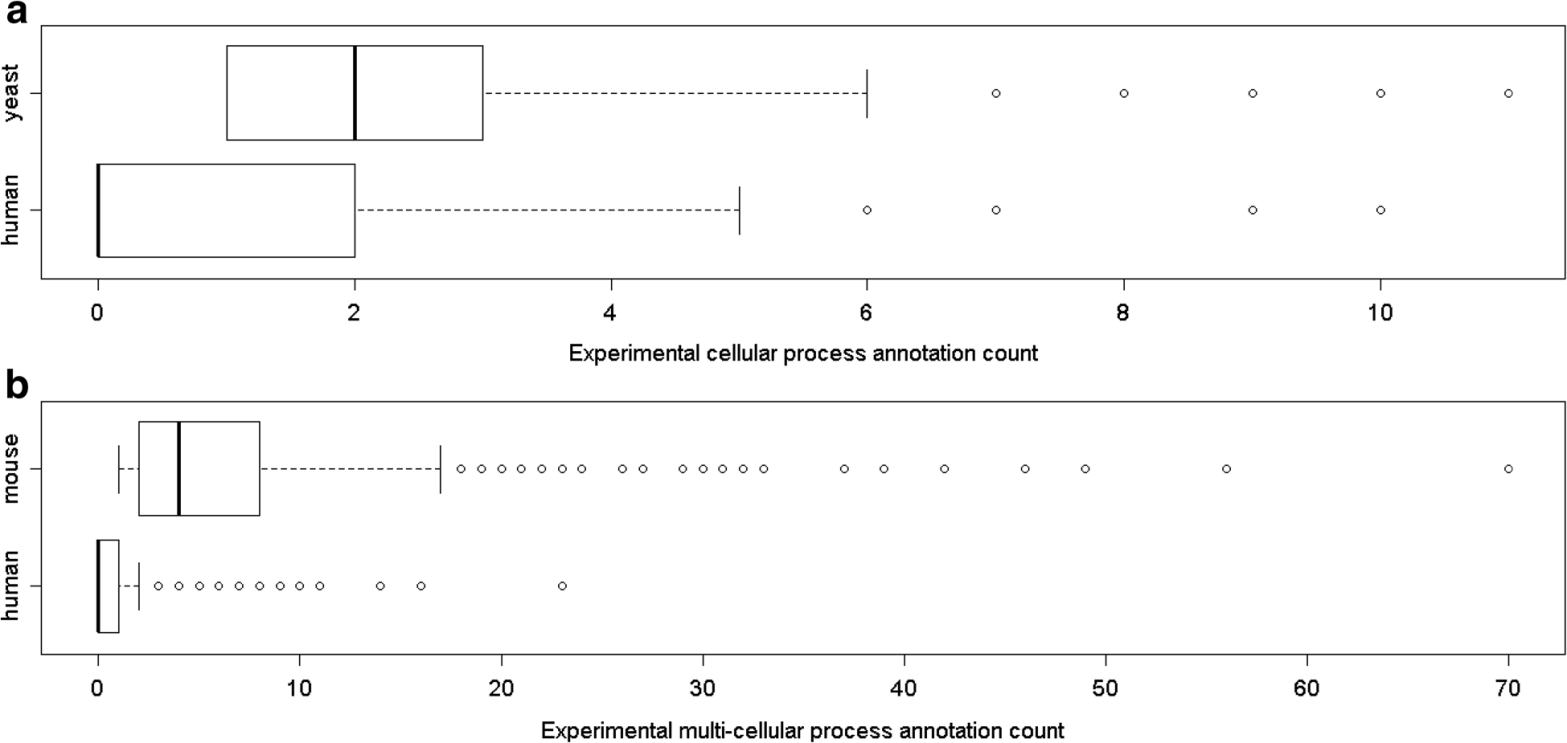
Distributions of the number of annotations for “incomplete” (actual human gene annotations) and “complete” (orthologs in yeast or mouse) annotation sets. **a** comparison between experimentally-supported GO annotations (cellular-level processes only) for human genes, compared to their orthologs in yeast, for well-studied yeast genes. **b** comparison between experimentally-supported GO annotations (multicellular organism-level processes only) for human genes, compared to their orthologs in mouse, for well-studied mouse genes

We attempted to quantify the incompleteness of current human experimental GO annotations, in order to make our study as relevant as possible to functional analysis of human genes. As this has not been done before, we opted for a straightforward approach: simply counting the number of annotations actually present in the GO knowledgebase for a human gene, and comparing it to the number of annotations expected if it were “completely” annotated. The difference between the number of actual annotations, and the number of expected (complete) annotations, gives a measure of incompleteness of the current experimental knowledge. Of course, we cannot know the number of expected annotations, so we estimated this number using a process described in detail in Methods. Briefly, we identify genes that have been well studied in a model system (yeast or mouse), and have a human ortholog, and consider them to be “completely” annotated. We then compare the number of annotations for each completely annotated gene to that of their human ortholog. We focus on GO biological process annotations; however, we recognize that GO biological processes span multiple levels of biological organization, so we consider separately GO cellular processes (using yeast as the best-studied model system) and GO multicellular organism-level processes (using mouse as the best-studied model system). Figure 2 shows the distribution of annotations for human genes, compared to their orthologs in yeast (for cellular processes, Fig. 2a) and mouse (for multicellular organism processes, Fig. 2b). It is evident from this plot that human experimental GO annotations are quite incomplete, with annotations for multicellular organism level processes being substantially more incomplete than for cellular level processes. We recognize that this method of estimating incompleteness of human annotations is a very rough approximation, as it assumes equivalence between annotations of different sub-branches and depths in the ontology. We mitigated this issue by first removing “redundant” GO annotations: if a gene is annotated to two GO terms where one term is an ancestor (using either is-a or part-of relations) of another, the less specific annotation is removed, as the more specific annotation also implies the less specific one. We note that our method is likely to underestimate of the actual incompleteness, since of course even well-studied genes are not completely studied or annotated. Nevertheless, it provides a rough estimate of the incompleteness of experimentally-supported human gene annotations, which we use to guide simulations of incomplete annotation sets (see Methods for details), in order to assess how incompleteness of human gene annotations can affect downstream analyses.

### The change of pairwise gene semantic similarities due to incomplete annotations

**Fig. 3:**
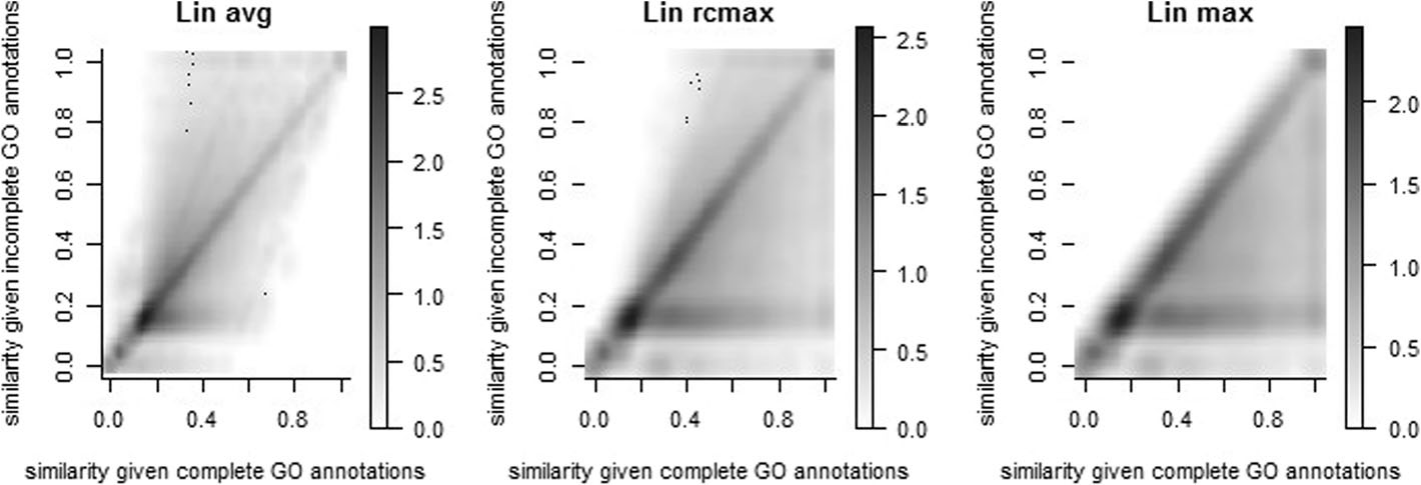
Pairwise gene semantic similarities for complete vs. incomplete cellular process annotations using Lin’s semantic similarity measure. Each point represents a unique gene pair with the value on X axis as their similarity for the complete annotations and the value on Y axis as their similarity for a random simulated incomplete set of annotations. Therefore, each gene pair is repeated 100 times in each plot, with each pair having the same similarity for complete annotations but a different similarity under a different simulated incomplete annotation set

Figure 3 shows how incompleteness affects the calculated pairwise gene similarities, for Lin’s similarity measure (other measures show similar effects, as displayed in Additional file 2: Figures S1 and S2). Each graph plots the similarity score of a pair of genes from an incomplete set (the graphs combine the results from all 100 simulated incomplete sets) vs. the score for that same pair in the complete set. Values along the diagonal indicate identical scores in the complete and incomplete sets, with values in the upper triangle indicating increases in similarity scores for incomplete annotations, and values in the lower triangle indicating decreases. Perhaps counter-intuitively, the pairwise gene similarity can either increase or decrease when annotations become incomplete, depending on the similarity measure and the gene pair. The effect can be very different for different measures, particularly depending on how a measure combines pairwise annotation similarities into a pairwise gene similarity. For example, scores obtained by averaging over all pairs of cellular process annotation similarities (Fig. 3, Lin AVG) can be either decreased or increased when annotations become incomplete, and tend to increase on average. This is simply because, even for two genes with identical GO annotations, the average similarity will decrease as the number of annotations increases. The average includes both matching (high similarity score) pairs, and non-matching (low-similarity score) pairs, and as the number of annotations increases the number of matching pairs grows much more slowly than the number of non-matching pairs: for N annotations there are N exactly matching pairs, but N(N-1)/2 mismatching pairs. Thus, the average score method depends on the number of annotations, which will severely limit its applicability. In contrast, scores obtained by averaging only those best-matched pairs of annotation similarities (Fig. 3, Lin BMA) are not affected by this dependency, and were much more likely to be decreased than increased by annotation incompleteness. Not surprisingly, scores using the maximum annotation pairwise similarity were always equal or decreased by annotation incompleteness (Fig. 3, Lin MAX). A similar pattern of change was observed for other similarity measures [Additional file 2: Figures S1 and S2]. Interestingly, for most similarity measures (except for JiangConrath, Cosine and WeightedJaccard measures), we observed a horizontal line of high density at a similarity value (given incomplete cellular annotation data) around 0.15–0.2 in most of these plots. This is due to the fact that for the incomplete annotation sets, roughly 25% of the pairwise distances (roughly between the 25th percentile and the 50th percentile of the distribution) fall in a narrow interval of roughly 0.15–0.2 (see Additional file 2: Figure S3), reflecting what is effectively a lower bound on the similarity score.

### Accuracy of gene clustering methods for “complete” annotation sets

**Fig. 4:**
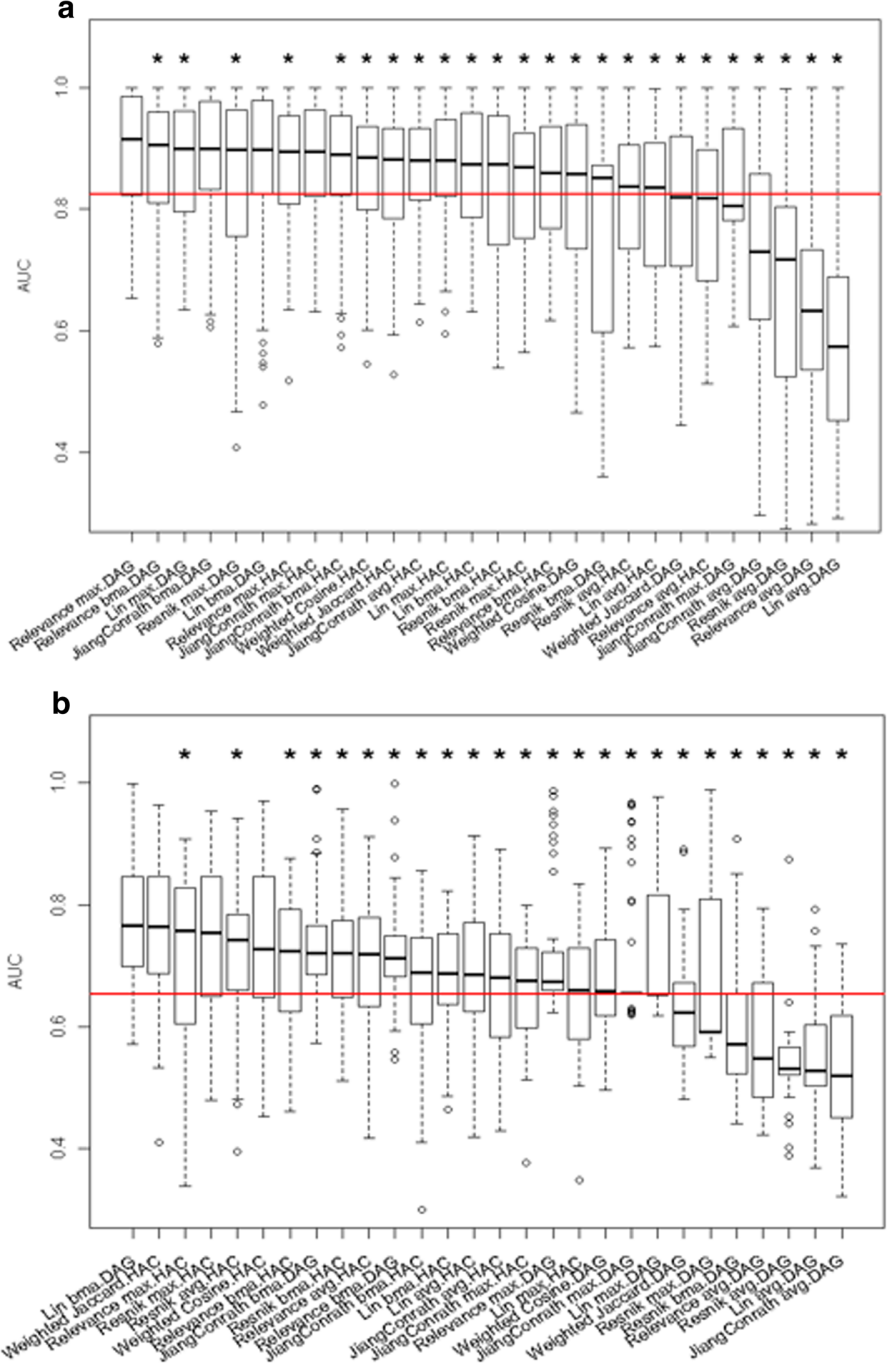
Distribution of AUC of gene-clustering using “complete” annotations. Panel (**a**) plots AUC of clustering using cellular process annotation set, and panel (**b**) plots AUC of clustering using multicellular organism process annotation set. HAG and DAG represent hierarchical clustering and Directed Acyclic Graph (CliXO) clustering, respectively. MAX, BMA and AVG represent the maximal functional similarity, the average of best-matched functional similarities, and the average of all functional similarities among genes, respectively. Combinations were ordered by the median AUC value. The red line represents the median AUC value across all combinations. An asterisk above a boxplot indicates that the AUC of the corresponding combination is significantly lower than the best (the combination with highest median AUC). The significance is determined by one-directional paired t test, P < 0.05

We first assessed the accuracy of different combinations of semantic similarity measure, and gene clustering method, in terms of recovering the known structure of the GO biological process classes (see section 2.5). We calculated the AUC for different clustering thresholds to compare the gene clusters obtained from the complete annotation sets, to the actual clusters from the relationships between GO terms (Fig. 4); an AUC of 1 indicates perfect clustering for that class. This may seem like a circular exercise, but it sets a base level for how well the results from each clustering method can capture the groupings that were present in the original input data. It will then allow us to see how accuracy is affected by incompleteness, as described in the next section below. For the “complete” cellular process annotation set (Fig. 4a), the performance of most measures is quite good, with more than 20 combinations having a median greater than 0.8.

Overall, for cellular processes, the performance tends to be better when two conditions hold: 1) the semantic similarity measure uses either maximal functional similarity between genes or the average-best-matched functional similarities between genes, and 2) the DAG clustering (CliXO) was applied. According to a one-directional paired t test, the combinations of Relevance MAX, JiangConrath BMA and Lin BMA utilizing DAG clustering, and combination of JiangConrath MAX utilizing HAC clustering, have significantly higher AUC than other combinations. The poor overall performance of similarity measures that average all pairwise annotation scores is not surprising given its dependence on the number of annotations, which varies across different genes as described above. The better overall performance of DAG clustering results from allowing genes to be grouped into multiple clusters, which is a key element of the Gene Ontology structure.

By contrast, the overall performance of gene clustering based on multicellular organism-level processes is quite poor (the overall median AUC value across all measures is below 0.7). This may be due to the fact that this annotation set has, on average, a much larger number of distinct annotations per gene than does the cellular process set (Fig. 2). If two genes work together in one or a few processes but not in others, their overall similarities will be low and they will not tend to be clustered together. In other words, information about conditional similarity in functions can be lost in the overall score, and therefore in the gene clusters constructed from these scores. According to one-directional paired t test, Lin BMA utilizing DAG clustering, and Resnik MAX, Weighted Jaccard and Weighted Cosine utilizing HAC clustering have significantly higher AUC than other combinations. In addition, the performance of DAG clustering decreases substantially for clustering using multicellular process annotations: three out of the top four combinations with significantly higher AUC for reconstructing cellular GO classes utilized DAG clustering (Fig. 4a); only one out of the top four combinations with significantly higher AUC for reconstructing multicellular GO classes utilized DAG clustering (Fig. 4b). This result is consistent with our interpretation that conditional similarities can be effectively lost in the overall pairwise score, so that the DAG clustering property of allowing multiple clusters for each gene is no longer an advantage when the diverse annotations are summarized by a single similarity score.

### Accuracy of gene clustering with incomplete annotations

**Fig. 5:**
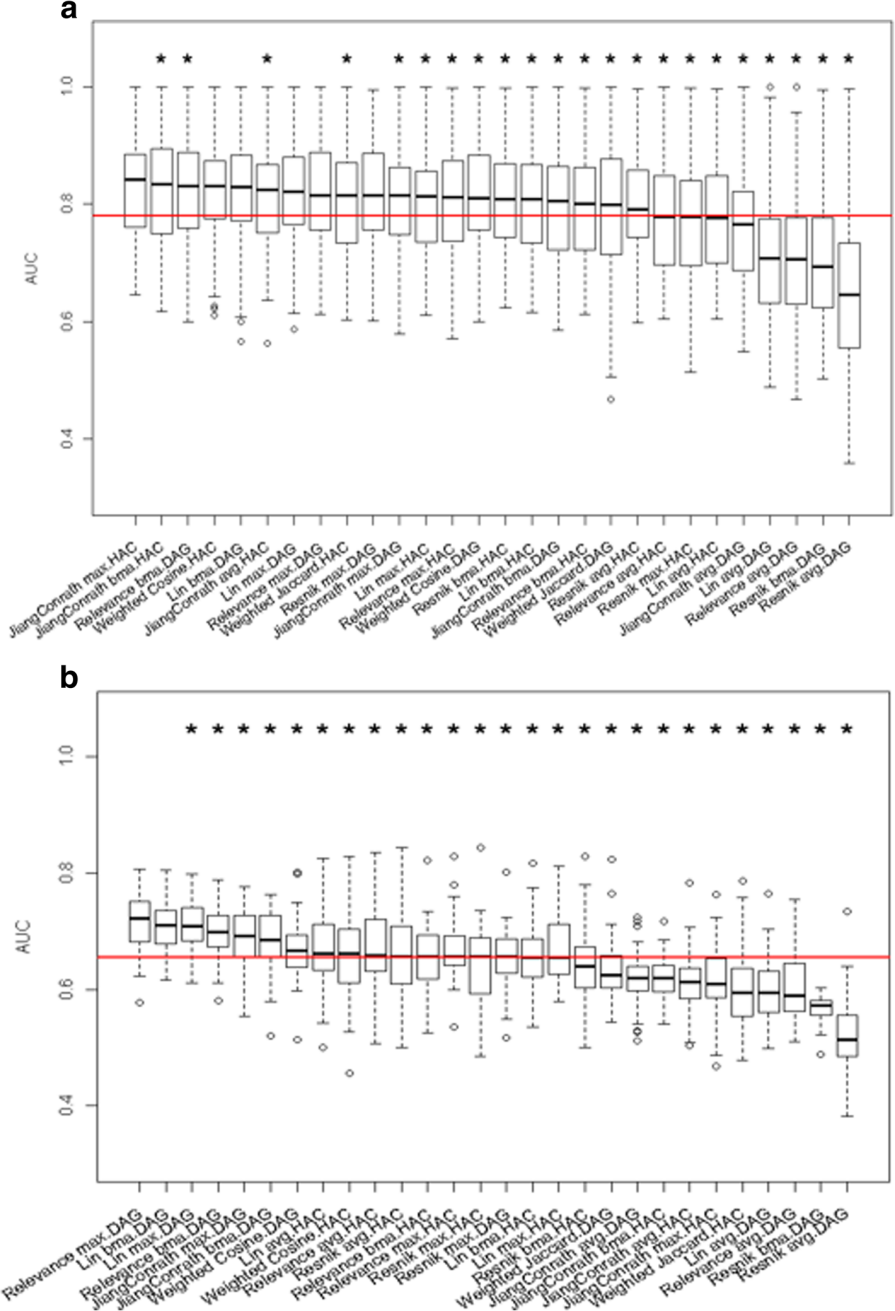
Distribution of AUC of gene-clustering using “incomplete” annotations. Panel (**a**) plots AUC of clustering using cellular process annotation set, and panel (**b**) plots AUC of clustering using multicellular organism process annotation set. HAG and DAG represent hierarchical clustering and Directed Acyclic Graph (CliXO) clustering, respectively. MAX, BMA and AVG represent the maximal functional similarity, the average of best-matched functional similarities, and the average of all functional similarities among genes, respectively. Combinations were ordered by the median AUC value. The red line represents the median AUC value across all combinations. An asterisk above a boxplot indicates that the AUC of the corresponding combination is significantly lower than the best (the combination with highest median AUC). The significance is determined by one-directional paired t test, *P* < 0.05

Not surprisingly, the clustering accuracy for “incomplete” annotation sets was lower than for “complete” annotation sets. For “incomplete” cellular process annotations, the median AUC value across all combinations decreases from 0.82 (Fig. 4a) to 0.78 (Fig. 5a).

For “incomplete” multicellular organism process annotation sets, while the median AUC value across all combinations is the same as for the complete set, the best combinations perform substantially worse on incomplete data, e.g. the Lin-BMA-DAG combination has an average AUC of 0.76 on complete data (Fig. 4b) with a maximum of 1 (perfect performance), while on incomplete data the average AUC is 0.72 with a maximum of 0.8 (Fig. 5b). The average performance of different combinations on multicellular processes is much worse than on cellular processes. Given the poor clustering results on even the complete multicellular process annotations as described above, this is not surprising.

In general, the best performing combinations under one set of conditions (cellular vs multicellular, complete vs incomplete) are not among the best performing combinations under another set of conditions. We identified the best performing combinations for complete and incomplete annotation sets, and both cellular and multicellular processes (Table 1).

**Table 1:** Best combinations of similarity and clustering methods for recovering the known structure of GO classes

Annotation completeness	Type of GO classes	Best combinations	
		Clustering methods	Semantic similarity measure
complete	cellular	HAC	JiangConrath MAX
		DAG	Relevance MAX, JiangConrath BMA, Lin BMA
	multicellular	HAC	Resnik MAX, Weighted Jaccard, Weighted Cosine
		DAG	Lin BMA
incomplete	cellular	HAC	JiangConrath MAX, Weighted Cosine
		DAG	Lin BMA, Lin MAX, Relevance MAX, Resnik MAX
	multicellular	DAG	Lin BMA, Relevance MAX

Only one combination, Lin BMA utilizing DAG clustering (CliXO), is among the top performing combinations in all cases, and JiangConrath MAX tends to perform best when utilizing hierarchical clustering. The top performing combinations never use the AVG method for combining similarity scores. Overall, a larger number of top performing combinations utilize DAG clustering.

To assess whether the accuracy calculations for our incomplete data sets were consistent between different simulated sets, we calculated the coefficient of variation (CV) of all AUC values (each simulated set has a corresponding AUC value) for each GO class. The distribution of AUC values for each measure/algorithm combination was then plotted as shown in Addtional file 2: Figure S4. Overall, there is a high degree of consistency: the grand median of CV is around 10%, i.e. on average there is an around 10% deviation of AUC value from the mean AUC value for each simulated set. Specifically, for simulated cellular process sets, for most combinations of measure and algorithm, CV values are narrowly distributed around 10% (except for Resnik-BMA-DAG, Resnik-AVG-DAG and Relevance-AVG-DAG). For simulated multicellular process sets, quite a few combinations gave more dispersed distribution of CV values with the 75th percentile close to 20%. This indicates a smaller degree of consistency for simulated multicellular process sets than the cellular process sets, though still showing overall consistency. This result is expected given the much higher degree of incompleteness of the multicellular process annotation sets compared to cellular processes (Fig. 2).

### Measure the change of clusters due to incomplete annotations

The preceding sections compared the clusters obtained for either complete or incomplete annotation sets, to the actual GO classes. We used this as a proxy for clustering *accuracy*. In this section, we compare the clusters obtained for a given method (combination of similarity measure and clustering algorithm) on the incomplete annotation sets, to those obtained on the complete annotation sets. Thus we are assessing the *robustness* of each method to incompleteness.

**Fig. 6:**
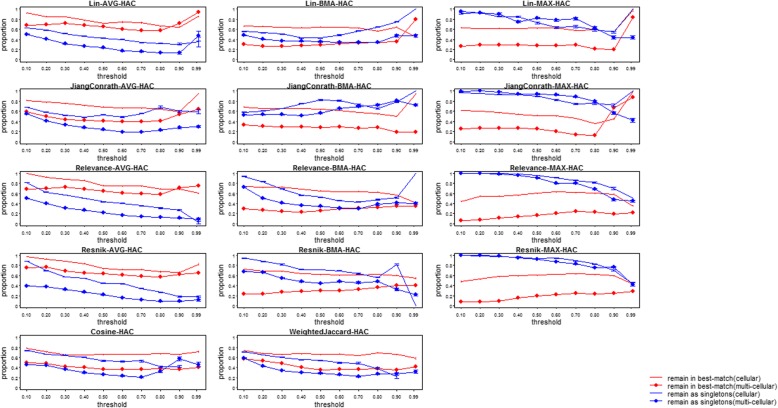
Plots of the robustness to annotation incompleteness for semantic similarity methods, for different similarity measures using hierarchical clustering. Points with filled circles show robustness of multicellular process annotation sets; lines without points show robustness of cellular process annotation sets. In red is the fraction of genes originally clustered together using complete annotations, that remained in the best-matched cluster using incomplete annotations. In blue is the fraction of singletons (unclustered genes) originally derived using complete annotations, that remained as singletons using incomplete annotations. A total of 10 different clustering thresholds increase from the left to the right evenly, based on the height of corresponding hierarchical tree from the leaves (0) to the root (1)

**Fig. 7:**
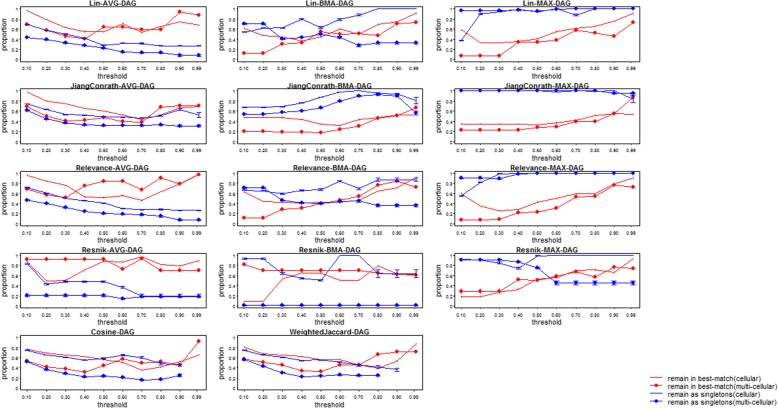
Plots of the robustness to annotation incompleteness for semantic similarity methods, for different similarity measures using DAG clustering. Points with filled circles show robustness of multicellular process annotation sets; lines without points show robustness of cellular process annotation sets. In red is the fraction of genes originally clustered together using complete annotations, that remained in the best-matched cluster using incomplete annotations. In blue is the fraction of singletons (unclustered genes) originally derived using complete annotations, that remained as singletons using incomplete annotations. A total of 10 different clustering thresholds increase from the left to the right evenly, based on the height of corresponding hierarchical tree from the leaves (0) to the root (1)

Figures 6 and 7 show the robustness of different similarity measures, gene-level scoring, and clustering method to incomplete data: specifically, the proportion of genes either remaining in the same (best-matching, see Methods above for details) cluster, or as singletons, using the “complete” and “incomplete” annotation sets. We determined clusters at various thresholds, values near 0 generate multiple, small clusters by cutting near the tips of the tree generated by clustering, while larger values create larger clusters by cutting nearer to the root. Overall, robustness to incompleteness was surprisingly high for most combinations, meaning that incompleteness did not result in extreme differences in the clusters. Nevertheless, the differences were substantial. For cellular processes, most combinations result in over half of the genes being clustered similarly in both the complete and incomplete sets (red lines in Figures 6 and 7). For multicellular organism processes, robustness was substantially smaller. The robustness estimates for each combination are very similar for different simulated incomplete sets (error bars in Figures 6 and 7). In general, combinations using the gene-level averaging method (AVG) were the most robust to incompleteness. This is perhaps not surprising, for the same reason (described above) that they result in low clustering accuracy: the pairwise gene similarities are averaged over a large number of pairwise annotation similarity scores, and removing some of these pairs has a smaller effect on the overall average than on the best-match average or maximal score. Best-match-average (BMA) combinations were somewhat less robust, with the exception of the Resnik measure, that was substantially less robust at lower clustering thresholds. The maximum (MAX) methods were generally the least robust to incompleteness, with the Resnik measure again having the smallest robustness at lower thresholds. For singletons (unclustered genes, blue lines in Figures 6 and 7), on the other hand, maximum score approaches tended to be the most robust, as genes with low maximum scores to all other genes in the complete annotation set will remain this way when annotations are removed.

## Discussion

We assessed the effects of annotation completeness on the distribution of pairwise gene semantic similarity scores, and subsequent effects on the clusters derived from these scores. We performed our assessments on all combinations of similarity measure and clustering method for recovering the known GO classes, using both “complete” and “incomplete” annotations. Specifically we considered 14 previously published similarity measures, and two types of clustering, hierarchical and CliXO. For both complete and incomplete annotation sets, measures which create a pairwise gene similarity by using the maximum or best matched average over all pairwise annotation similarities tend to perform best. In addition, the CliXO clustering method, combined with appropriate similarity measures, tends to perform better than hierarchical clustering. A few particular methods, such as Lin BMA and Relevance MAX utilizing CliXO, are generally among the most accurate for both complete and incomplete annotation sets, and both cellular and multicellular organism processes (Table 1). The best-match-average method of deriving gene-level scores, however, generally shows greater robustness to incompleteness than maximum method, meaning that the cluster identities are more similar to those obtained for “complete” annotations. Therefore this method might be preferable for many clustering applications. The averaging method at the gene-level, while the most robust, has much lower clustering accuracy than any other method. This is at least in part because the signal of similar annotations (shared between two genes) is diluted to varying degrees by the noise of dissimilar annotations, an effect that depends on the number of annotations.

We find that hierarchical agglomerative clustering approaches (which yield only strict hierarchies, i.e. a cluster can have only one parent cluster) have higher accuracy with similarity measures that utilize the maximum pairwise annotation score, or with the WeightedJaccard or WeightedCosine measures; the WeightedJaccard or WeightedCosine measures are more robust to incompleteness. The CliXO clustering method, because it can allow multiple parent clusters, is able to utilize information from multiple different annotations captured in the best-match average scores (which average over the best match between each annotation of one gene, and an annotation of the other gene). This is consistent with the testing of CliXO with best-match-average scores by Kramer et al. [25] (though they utilized the Resnik measure, which we find to be less accurate, and less robust to incompleteness than some other measures). However, when the number of distinct annotations for each gene is too large, such as our multicellular process annotation sets, this advantage disappears.

We find that while several combinations of similarity measure and clustering algorithm perform well for representing GO cellular processes, all combinations perform much worse for representing multicellular organism-level processes. This likely reflects the greater complexity of this branch of the GO biological process ontology, and the larger number of annotations in both the complete and incomplete sets (and therefore the greater loss of information when reducing into one dimension of a similarity score).

## Conclusions

Our study has attempted to estimate a lower bound on the incompleteness of experimental GO annotations of human genes, by comparing with experimental annotations of orthologous genes in highly studied model organisms (yeast and mouse). We find that human annotations are highly incomplete, and much more incomplete for multicellular organism level processes than for cellular level processes. We also find, not surprisingly, that genes tend to be more highly pleiotropic (fewer distinct annotations per gene) at the multicellular level, than at the cellular level. We used this estimate to simulate incomplete annotation sets, and assess how this incompleteness can affect downstream GO-based analyses, specifically pairwise semantic similarity scores and gene similarity clusters derived from them. To make this comparison, we also needed to assess the clusters derived from “complete” annotation sets. We find that for cellular-level process annotations, which are moderately incomplete and show less functional pleiotropy, the DAG-based CliXO clustering method performs well with several different GO term semantic similarity measures. However, because genes are generally annotated to multiple, distinct terms, it is critical that the overall gene pairwise similarity is derived from a method that attempts to first match up each GO annotation for one gene with its cognate for the other gene (either using the maximum method, or best-match-average method), rather than taking a simple average over all possible matches (the average method). For multicellular processes, for which genes display much greater pleiotropy, nearly all combinations of similarity measures and clustering methods perform relatively poorly, on both complete and incomplete annotation sets, at least in part due to the difficulty in reducing a comparison over a large number of distinct functional annotations to a single gene-gene similarity score. However, in all cases, we find a substantial decrease in both clustering accuracy and robustness when annotations are incomplete, underscoring the importance of increasing the completeness of GO annotations for supporting computational analyses of gene function.

## Methods

### Creating a representation of a set of “completely annotated” human genes

We first created an approximation to a set of human genes that are “completely annotated” with respect to GO biological process terms. Recognizing that yeast is the best-studied eukaryotic cellular system, and mouse the best-studied vertebrate animal, we determined two sets separately: cellular processes, and multicellular organism-level processes. For cellular processes, we considered all yeast genes that are associated with at least 75 distinct publications in PubMed to be “well studied” experimentally. Similarly, for multicellular processes, we considered all mouse genes that are associated with at least 75 distinct publications. Associations between PubMed IDs and yeast genes and mouse genes were obtained from *Saccharomyces* Genome Database (SGD) [30] and the Mouse Genome Informatics (MGI) Data and Statistical Reports [31], respectively. This resulted in a set of 866 “well-annotated” yeast genes, and a set of 850 “well-annotated” mouse genes. All GO annotations having experimental evidence codes (EXP, IDA, IPI, IGI, IEP, IMP) were extracted for each yeast/mouse gene in the final sets, from the GO database (AmiGO 2.0, Mar 12, 2014). We used the Bioconductor package GO.db 2.8.0 to remove “redundant” annotations; this included annotations to the same term using a different piece of evidence, and annotations to less specific terms that were already covered by a more specific annotation.

To convert these model organism annotations to an approximation of “complete” human gene annotations, each yeast (cellular processes) or mouse (multicellular organism processes) gene was mapped to the corresponding least-diverged human ortholog, as defined from PANTHER [3]. Of the 866 well-annotated yeast genes, and 850 well-annotated mouse genes, 434 and 813, respectively, could be mapped to least-diverged orthologs in humans. We took these sets as our approximated “completely annotated” human gene sets for cellular processes (434 human genes) and multicellular organism processes (813 human genes).

### Quantify incompleteness of experimental biological process annotations among human genes

**Fig. 8:**
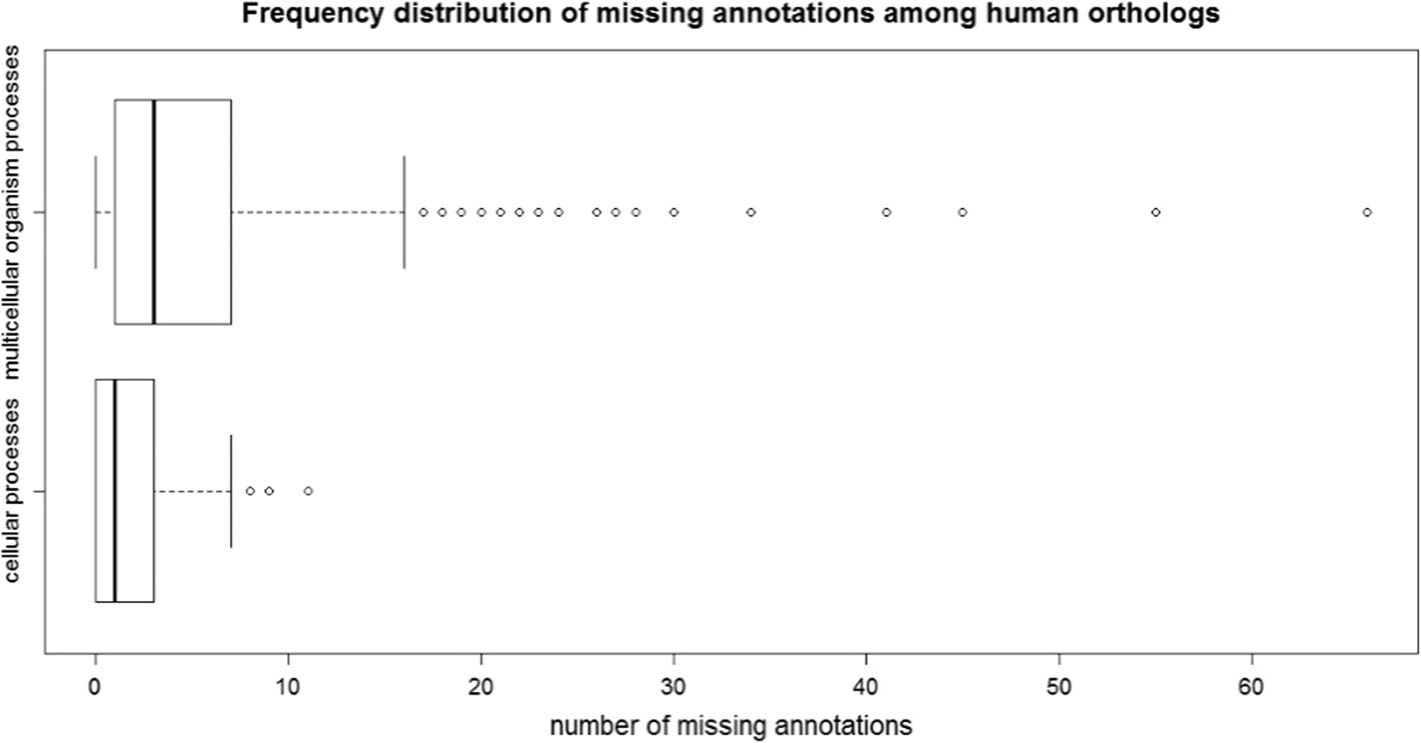
Frequency distribution of missing annotations among human genes. The x-axis represents the number of missing annotations of each human gene compared with its “well-annotated” ortholog in yeast (cellular processes) or mouse (multicellular organism processes). The degree of incompleteness of human gene annotation is much greater for multicellular processes than cellular processes

For these two sets of approximated “completely annotated” human genes, we compared the number of GO annotations to the actual number of experimental annotations available for each of these genes. The difference between these numbers gives an approximation to the incompleteness of experimental human gene annotations. We opted for a simple count difference of annotations rather than a more detailed comparison, as even the well-studied yeast and mouse genes are incompletely annotated. Thus, a simple count difference provides a conservative estimate of incompleteness, which is appropriate for our analysis. The distributions of annotation count differences between the “complete” and actual annotation sets are shown in Fig. 8 (again for simplicity, all differences less than 0, i.e. where the human ortholog has more GO annotations than its corresponding ortholog in either yeast or mouse, are set to 0).

### Simulating “incompletely annotated” genes

We implemented a random sampling procedure to generate a large number of random, incomplete annotation sets. This procedure randomly removes annotations from the “complete” cellular process annotation set and the “complete” multicellular organism process annotation set (section 4.1), according to the distributions in Fig. 8. The procedure for incomplete annotation set simulation was as follows. Let ***N*** represent the discrete set of numbers of missing annotations for each orthologous human gene, and let ***n*** represent a specific value from that set (i.e. 0<***n***<70). ***N*** is ordered from largest to smallest number of missing annotations. For each member ***n*** in the set ***N***, let ***g***_***n***_ be the number of genes that have ***n*** missing annotations. Let ***S*** be the set of genes.

- Step 1: Select the number of annotations to remove from a completely annotated gene; begin with the largest remaining number of missing annotations, i.e. choose ***n*** = max (***N***).
- Step 2: from all remaining genes (i.e. with unmodified annotations) in ***S***, randomly select ***g***_***n***_ of them, denoted by ***s*** (genes in ***s*** must have at least ***n*** annotations)
- Step 3: randomly remove ***n*** annotations from each gene in ***s***
- Step 4: exclude ***s*** from ***S*** and exclude ***n*** from ***N***
- Step 5: repeat Steps 1-4 until complete.

The simulation was repeated 100 times to generate 100 different incomplete sets each of cellular and multicellular annotations. Gene-cluster analysis using “complete” and “incomplete” annotation sets were compared with each other to evaluate the impact of incompleteness of annotations.

### Calculation of GO-based similarities and clustering of genes using complete and incomplete annotation sets

For both complete and incomplete annotation sets, only those genes with at least one annotation were included in analysis. GO-based gene-gene similarity scores were first calculated. Information content (IC) based semantic similarity measures included Resnik's, Lin's, Jiang and Conrath's and Schlicker’s measures, and non-IC based measures included weighted Cosine and weighted Jaccard measures. For each IC based semantic similarity measure, three different methods were used to calculate pairwise annotation similarity scores to pairwise gene similarity scores: *average* of pairwise annotation similarities, *maximum* of annotation similarities and *best-matched* annotation similarities. Several R-based tools have been developed recently for computing both IC based and non-IC based semantic similarities. Specifically in this study, we used an R-based tool called csbl.go to calculate all similarities listed above. With csbl.go, a similarity score between genes can be automatically computed. For IC-based similarities, the probability of each GO term occurring in the set of annotations for all genes for different species is calculated by this tool. This information can be directly transformed to an IC value for each GO term. Thus, the only parameters need to be specified for calculating similarities are the name of the species, ontology domain and the name of similarity measure used for the calculation [27]. For each similarity measure, both hierarchical agglomerative clustering (HAC) and Directed Acyclic Graph (DAG) clustering were performed to cluster genes, separately for each annotation set (2 complete sets and 200 incomplete sets). The agglomerative nesting algorithm implemented in the R package ‘agne’ was used for hierarchical clustering; the Clique Extracted Oncology (CliXO) algorithm developed by Kramer et al. [25] was used for DAG clustering.

### Measuring the accuracy of clustering

**Fig. 9:**
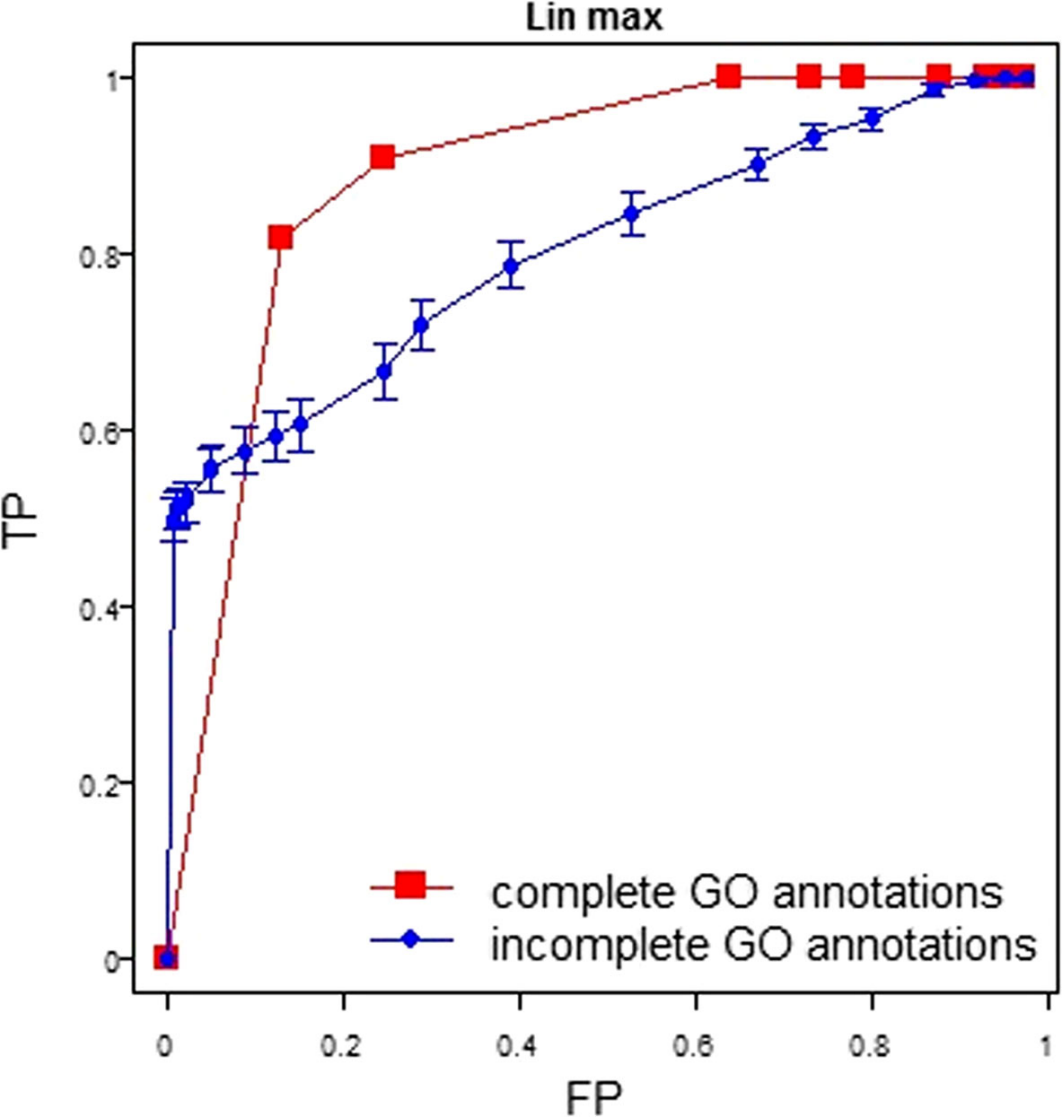
ROC of clustering of Macro-autophagy genes using Lin MAX measure and hierarchical clustering. The x-axis represents false positive rate, i.e. the proportion of other genes clustered into macro-autophagy gene set. The y-axis represents true positive rate, i.e. the proportion of macro-autophagy genes in macro-autophagy gene cluster. Each point corresponds to a different clustering threshold. The ROC is plotted for complete annotations and incomplete annotations (average and standard deviation shown for 100 randomly generated sets), respectively

We assessed the accuracy of clustering results by asking how well genes in the same GO class were clustered together. For each GO class with a meaningful size (containing between 5 and 50 genes in the given “complete annotation” set), we calculated a Receiver Operating Characteristic (ROC) curve that describes the true positive rate (TP, the proportion of genes from that class that are successfully clustered together) as a function of false positive rate (FP, the proportion of genes from other classes that are misclassified into that same cluster) for different clustering thresholds. TP and FP were calculated under 10 equally spaced thresholds of clustering. For each ROC curve, the area under the curve (AUC) was calculated. A perfect clustering would have an AUC of 1 for all GO classes, i.e. that all members of the class are clustered together before non-members are added to the cluster. An example ROC curve is shown in Fig. 9.

### Measuring the consistency of clustering from different simulated “incomplete” gene sets

We assessed the consistency of analysis from different simulated sets by calculating the CV(%) of AUC for clustering genes from the same GO class using different simulated sets: For each GO class with a meaningful size (containing between 5 and 50 genes in the given “complete annotation” set), we calculated the AUC as described above for each of simulated data sets. We then calculated the CV(%) of AUC (i.e. standard deviation of AUC values across all simulated set divided by the mean of AUC values) for each GO class, given each similarity measure and clustering algorithm. The distribution of CV(%) across all GO classes were calculated. A small CV(%) indicates a small data deviation from the mean AUC, which means a high degree of consistency of results between different simulated data sets.

### Measuring the robustness of clustering to incompleteness

**Fig. 10:**
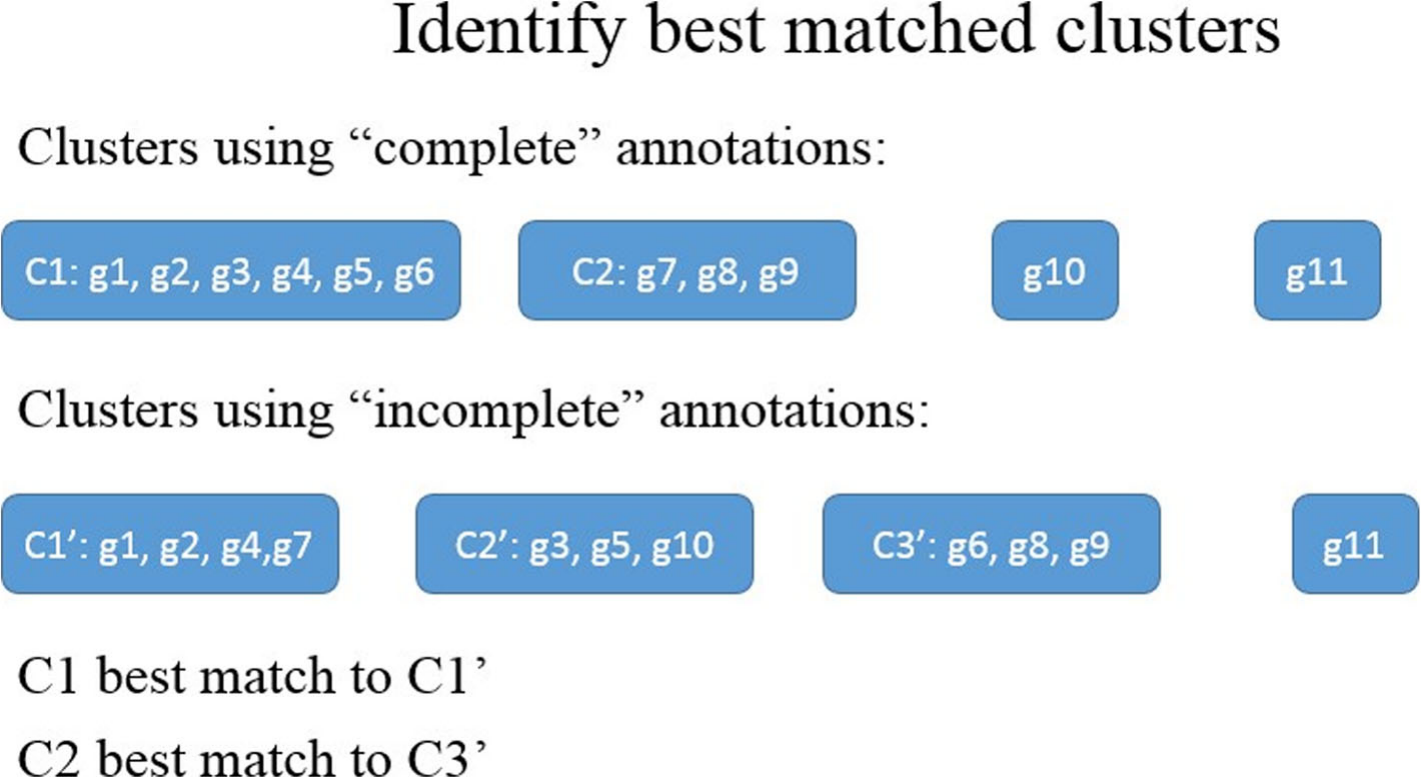
An example of determining matches between clusters of “completely annotated” genes and “incompletely annotated” genes. 10 different genes g1-g10 were clustered into C1 and C2 given complete annotations, and C1’, C2’ and C3’ given incomplete annotations. According to the number of overlapped genes, both C1’ and C2’ match to C1, and C3’ match to C2. Because C1 has more overlapping genes with C1’, C1 is the best match to C1’

We assessed the robustness of clustering to incompleteness by comparing how the clusters changed for incomplete relative to complete annotation sets. For each of the 10 clustering thresholds, gene clusters were obtained for all complete and incomplete annotation sets. For each threshold, and each incomplete annotation set, clusters were compared to the clusters obtained for the corresponding complete annotation set. This requires finding the best match between each cluster obtained from the complete annotation set, and a cluster obtained from the incomplete annotation set. The reciprocal best match clusters were determined as follows (Fig. 10):

1. Each cluster of “incompletely annotated” genes was matched to the cluster(s) of “completely annotated” genes with the largest number of overlapping genes.
2. Following step 1, if a cluster of “completely annotated” genes has multiple matched clusters of “incompletely annotated” genes, the one with the largest number of overlapping genes was considered as the best match.

For each cluster from the completely annotated set, we calculated the proportion of genes that were found in the best-matched cluster from an incompletely annotated set. For unclustered singletons from the completely annotated set, we calculated the proportion of genes that remained as singletons in the clustered incompletely annotated set.

## Additional files

Additional file 1: Introduction to gene-gene similarity measures (DOCX 89 kb)

Additional file 2: **Figure S1** Plots of pairwise gene similarity for complete vs. incomplete GO cellular process annotations. **Figure S2** Pairwise gene semantic similarities for complete vs. incomplete GO multicellular organism level process annotations. **Figure S3** Distribution of pairwise gene similarity scores for simulated incomplete annotation sets. For most measures, a large fraction (~ 25%) of these values lie in a very narrow range. **Figure S4** Distribution of the coefficient of variation for accuracy over 100 simulated incomplete annotation sets (see Fig. 5) (DOCX 778 kb)

## Abbreviations

AUC: Area under the curve
AVG: Gene-gene similarity score derived by averaging over all annotation similarities
BMA: Gene-gene similarity score derived by best-match-average
CliXO: Clique extracted ontology
DAG: Directed acyclic graph
FP: False positive
GO: Gene ontology
HAC: Hierarchical agglomerative clustering
IC: Information content
MAX: Gene-gene similarity score derived by maximum score match
ROC: Receiver operating characteristic
TP: True positive
