# Supplementary material for: GO functional similarity clustering depends on similarity measure, clustering method, and annotation completeness
Source: BMC Bioinformatics. 2019 Mar 27;20:155. doi: 10.1186/s12859-019-2752-2 (PMC6437941; doi:10.1186/s12859-019-2752-2)
Supplement: Supplementary file 1 — Introduction to gene-gene similarity measures (DOCX 89 kb) [file 12859_2019_2752_MOESM1_ESM.docx]

Additional file 1: introduction to gene-gene similarity metrics

Meng Liu^1^ and Paul D. Thomas^1. *^

^1^Department of Preventive Medicine, Keck School of Medicine, University of Southern California

*To whom correspondence should be addressed.

# IC-based semantic similarity metrics

In this study, csbl.go (1.4.0 version) was used to measure semantic gene-gene similarity. GO terms GO:0036302, GO:0036303, GO:0086094, GO:0097324, GO:1900164, GO:0036205, GO:0036228, GO:0036297, GO:0097301 and GO:1900622 were unavailable in csbl.go, and were therefore excluded from our analysis. Four measures we considered in this study require calculating the “information content” (IC) of GO terms. The IC of a term *t_i_* is defined by:

$IC\left(t_{i}\right)=-\log\left(p\left(t_{i}\right)\right)$ (1)

where *p*(*t_i_*) is the probability of the term *t_i_* occurring in the set of annotations for all genes in the GO database:

$p\left(t_{i}\right)=\frac{freq\left(t_{i}\right)}{freq\left(t_{root}\right)}$ (2)

where *t_root_* represents the root term of ontology,$freq\left(t_{root}\right)$ equals the total number of gene annotations in that aspect of the ontology and$freq\left(t_{i}\right)$ equals the number of genes annotated with the term *t_i_* or any of its descendant terms (subclasses). The more specific and informative a term is, the fewer genes are annotated by that term. Intuitively, as we move from the highest root term to the lowest leaf terms,$freq\left(t_{i}\right)$ decreases and *IC*(*t_i_*) increases (from 0 to infinity). This makes IC a measure of how much biological information a GO term imparts, which can be further applied to semantic similarity measures.

The most common IC-based semantic similarity measures using GO are Resnik's, Lin's, Jiang and Conrath's, and Schlicker’s measures. Resnik’s measure [1] simply uses the IC value of the common ancestors of two terms as their similarity, and is denoted by:

$Sim_{\mathrm{Re}s}\left(t_{1},t_{2}\right)=IC\left(LCA\left(t_{1},t_{2}\right)\right)$ (3)

Where $LCA\left(t_{1},t_{2}\right)$ represents the lowest (most specific) common ancestor term in the ontology that is shared by terms *t*_1_and *t*_2_. The other 3 measures, however, take into consideration both the IC values of terms *t*_1_ and *t*_2_ and the IC value of$LCA\left(t_{1},t_{2}\right)$.

Lin's measure [2] takes the similarity between terms *t*_1_ and *t*_2_ as the ratio between the IC value of their LCA and the average IC value of those two single terms:

$Sim_{Lin}\left(t_{1},t_{2}\right)=\frac{2IC\left(LCA\left(t_{1},t_{2}\right)\right)}{IC\left(t{}_{1}\right)+IC\left(t_{2}\right)}$ (4)

Jiang and Conrath proposed a measure [3] that is a function of the difference between the average IC value of two terms and the IC value of the ICA they share:

$Sim_{Jiang}\left(t_{1},t_{2}\right)=\frac{1}{\left(IC\left(t_{1}\right)+IC\left(t{}_{2}\right)-2IC\left(LCA\left(t_{1},t_{2}\right)\right)+1\right)}$ (5)

Both $Sim_{Lin}\left(t_{1},t_{2}\right)$*and* $Sim_{Jiang}\left(t_{1},t_{2}\right)$ are determined by how “similar” each term (*t*_1_ and *t*_2_) is to the LCA of those terms. $Sim_{\mathrm{Re}s}\left(t_{1},t_{2}\right)$, on the other hand, depends on the specificity of the LCA itself. Schlicker developed a new measure [4] that combines both of these attributes by weighting Lin’s measure according the specificity of the *LCA* of t_1_ and t_2_, which he called the Relevance measure:

$Sem_{\mathrm{Re}l}\left(t_{1},t_{2}\right)=2\left(1-p\left(LCA\left(t_{1},t_{2}\right)\right)\right)\cdot\frac{IC\left(LCA\left(t_{1},t_{2}\right)\right)}{\left(IC\left(t_{1}\right)+IC\left(t_{2}\right)\right)}$ (6)

In contrast to $Sim_{\mathrm{Re}s}\left(t_{1},t_{2}\right)$ which ranges between 0 and infinity*,*$Sim_{Lin}\left(t_{1},t_{2}\right)$*,* $Sim_{Jiang}\left(t_{1},t_{2}\right)$ and $Sim_{\mathrm{Re}l}\left(t_{1},t_{2}\right)$ *all* range between 0 and 1.

IC-based scores between GO terms given by each measure above can be used to calculate IC-based scores between gene products in three different ways, which we call average (avg), maximum (max) and best-match-average (bma), as described below [5-7]. Because each gene product can have multiple GO annotations, we need to calculate a *total similarity score* for gene $g_{1}$(annotated with $n$terms) vs. gene $g_{2}$(annotated with$m$terms), from the $n\times m$GO term similarity matrix$S$. Each element $s_{ij}$represents the similarity between the $i^{th}$ GO term of $g_{1}$and $j^{th}$ GO term of$g_{2}$. The similarity between $g_{1}$ and $g_{2}$ can be calculated in 3 ways as follows:

1. use the average score across all pairs of GO terms:

$Sim\left(g_{1},g_{2}\right)=\frac{\sum_{j=1}^{m} \sum_{i=i}^{n} s_{ij}}{mn}$ , (7)

1. Use the maximum score across all pairs of GO terms:

$Sim\left(g_{1},g_{2}\right)=\max\left\{s_{ij}:1\leq i\leq n,1\leq j\leq m\right\}$ (8)

1. Map each GO term of the gene product$g_{1}$to its best-matching term (i.e. the single, annotation with the highest similarity score) of gene product$g_{2}$, and then average scores of all these best-match mappings from$g_{1}$to$g_{2}$, and vice versa (i.e. mapping each annotation for $g_{1}$ to its best-match annotation for$g_{2}$. The larger average (either $g_{1}$to$g_{2}$, or $g_{2}$ to $g_{1}$) is used as the final score between $g_{1}$and$g_{2}$. The formula is defined as below:

$Scorecolumn=\frac{\sum_{j=1}^{m} \max\left\{s_{ij}:1\leq i\leq n\right\}}{m}$ (9)

$Scorerow=\frac{\sum_{i=1}^{n} \max\left\{s_{ij}:1\leq j\leq m\right\}}{n}$ (10)

The similarity between $g_{1}$and$g_{2}$*is then defined as:*

$Sim\left(g_{1},g_{2}\right)=\max\left\{Scorecolumn,Scorerow\right\}$ (11)

These three methods are referred as the “average”, “maximum”, and “best-match-average (bma)” in this paper. Therefore, a total of 12 IC-based similarities between genes are considered: Lin, JiangConrath, Resnik and Relevance measure, each with average, maximum and bma method.

# Vector-based similarity metrics

In addition to IC-based measures, we consider as well the Cosine and weighted Jaccard measures. The Cosine measure [8] is based on a vector model space: For a gene$g$, a vector$v=\left(w_{1},w_{2},\ldots,w_{n}\right)$ is built, with $w_{i}$ equal to $IC\left(t_{i}\right)$ if $g$ is annotated with term $t_{i}$ and 0 if not. The Cosine Similarity between two genes *g_i_* and *g_j_* is then defined as:

$Sim\left(g_{1},g_{2}\right)=\frac{v_{i}\cdot v_{j}}{\left|v_{i}\right|\left|v_{j}\right|}$ (12)

Where · represents the dot product and represent the vector norm.

The weighted Jaccard measure [9] is based on the vector model space as well: considering genes $g_{1}$and$g_{2}$annotated with term sets GO_1_ and GO_2_, the weighted Jaccard measure similarity between $g_{1}$and$g_{2}$is defined as:

(13)

# Distance between genes

Let $Sim\left(g_{1},g_{2}\right)$ denote similarity between two genes $g_{1}$and$g_{2}$, the corresponding distance between $g_{1}$and$g_{2}$is then defined by$d\left(g{}_{1},g_{2}\right)=1-Sim\left(g_{1},g_{2}\right)$. For Resnik’s similarity which ranges between 0 and infinity, $d_{\mathrm{Re}s}\left(g_{1},g_{2}\right)=\frac{1}{1+Sim_{\mathrm{Re}s}\left(g_{1},g_{2}\right)}$ [10].

# References

1. Resnik P. Using information content to evaluate semantic similarity in a taxonomy. In: In Proceedings of the 14^th^ International Joint Conference on Artificial Intelligence; 1995. p. 448-453

2. Lin D. An information-theoretic definition of similarity. In: In Proceedings of the 15th International Conference on Machine Learning. San Francisco, CA: Morgan Kaufmann; 1998. p. 296–304.

3. Jiang J, Conrath DW. Semantic Similarity Based on Corpus Statistics and Lexical Taxonomy. In: Proc of 10^th^ International Conference on Research in Computational Linguistics, ROCLING’97. Taiwan; 1997.

4. Schlicker A, Albrecht M. FunSimMat: a comprehensive functional similarity database. Nucleic Acids Res. 2008;36(Database issue):D434–D439. doi: 10.1093/nar/gkm806.

5. Lord PW, Stevens R, Brass A, Goble C. Investigating semantic similarity measures across the Gene Ontology: the relationship between sequence and annotation. Bioinformatics. 2003;19(10):1275–1283.

6. Schlicker A, Domingues F, Rahnenfu¨hrer J, Lengauer T. A new measure for functional similarity of gene products based on Gene Ontology. BMC Bioinformatics. 2006;7:302. doi:10.1186/1471-2105-7-302.

7. Sheehan B, Quigley A, Gaudin B, Dobson S. A relation based measure of semantic similarity for Gene Ontology annotations. BMC Bioinformatics. 2008;9:468. doi: 10.1186/1471-2105-9-468.

8. Bodenreider O, Aubry M, Burgun A. Non-lexical approaches to identifying associative relations in the gene ontology. Pac Symp Biocomput. 2005;p. 91–102.

9. Pesquita C, Faria D, Falc˜ao A, Lord P, Couto F. Semantic similarity in biomedical ontologies. PLoS Comput Biol. 2009;5(7):e1000443. doi: 10.1371/journal.pcbi.1000443.

10. Ovaska K, Laakso M, Hautaniemi S. Fast gene ontology based clustering for microarray experiments. BioData Min. 2008;1(1):11. doi: 10.1186/1756-0381-1-11.
