# Supplementary material for: GO functional similarity clustering depends on similarity measure, clustering method, and annotation completeness
Source: BMC Bioinformatics. 2019 Mar 27;20:155. doi: 10.1186/s12859-019-2752-2 (PMC6437941; doi:10.1186/s12859-019-2752-2)
Supplement: Supplementary file 2 — Figure S1 Plots of pairwise gene similarity for complete vs. incomplete GO cellular process annotations. Figure S2 Pairwise gene semantic similarities for complete vs. incomplete GO multicellular organism level process annotations. Figure S3 Distribution of pairwise gene similarity scores for simulated incomplete annotation sets. For most measures, a large fraction (~ 25%) of these values lie in a very narrow range. Figure S4 Distribution of the coefficient of variation for accuracy over 100 simulated incomplete annotation sets (see Fig. 5) (DOCX 778 kb) [file 12859_2019_2752_MOESM2_ESM.docx]

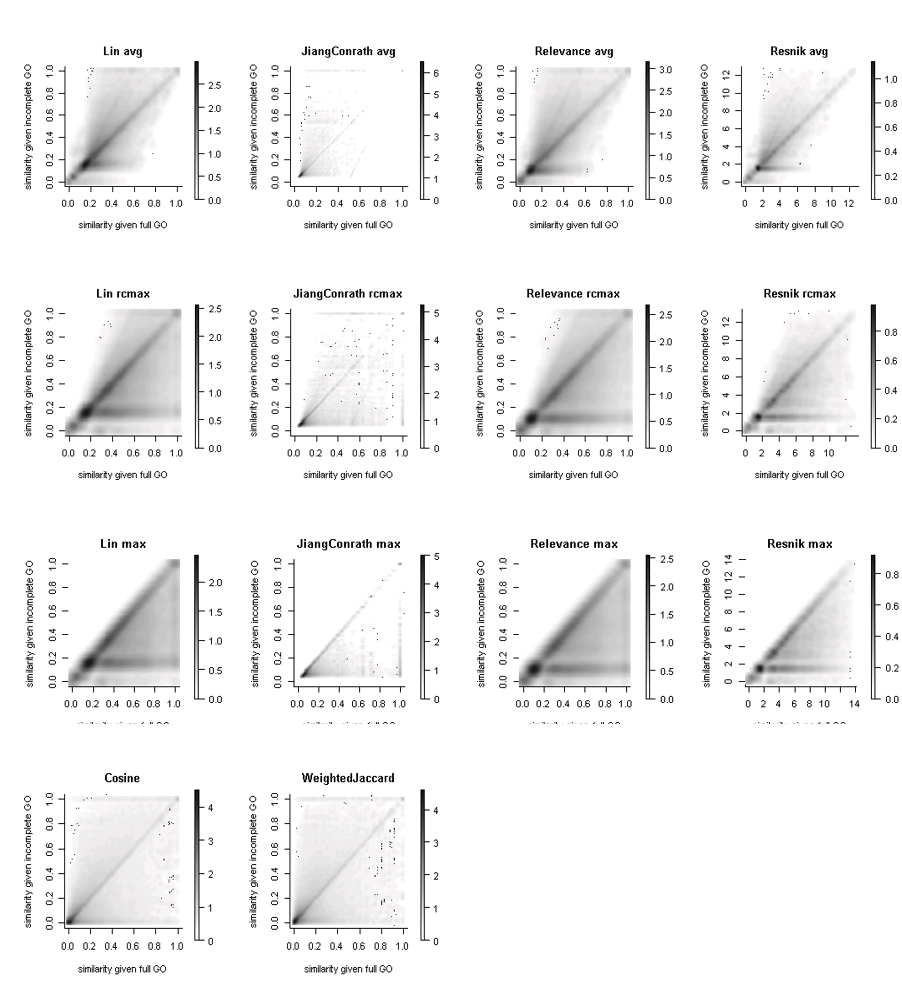


**Figure S1. Plots of pairwise gene semantic similarities for complete vs. incomplete GO cellular process annotations.** Each point represents a unique gene pair with the value on X axis as their similarity for the complete annotations and the value on Y axis as their similarity for a random simulated incomplete set of annotations. Therefore, each gene pair is repeated 100 times in each plot, with each pair having the same similarity for complete annotations but a different similarity under a different simulated incomplete annotation set.


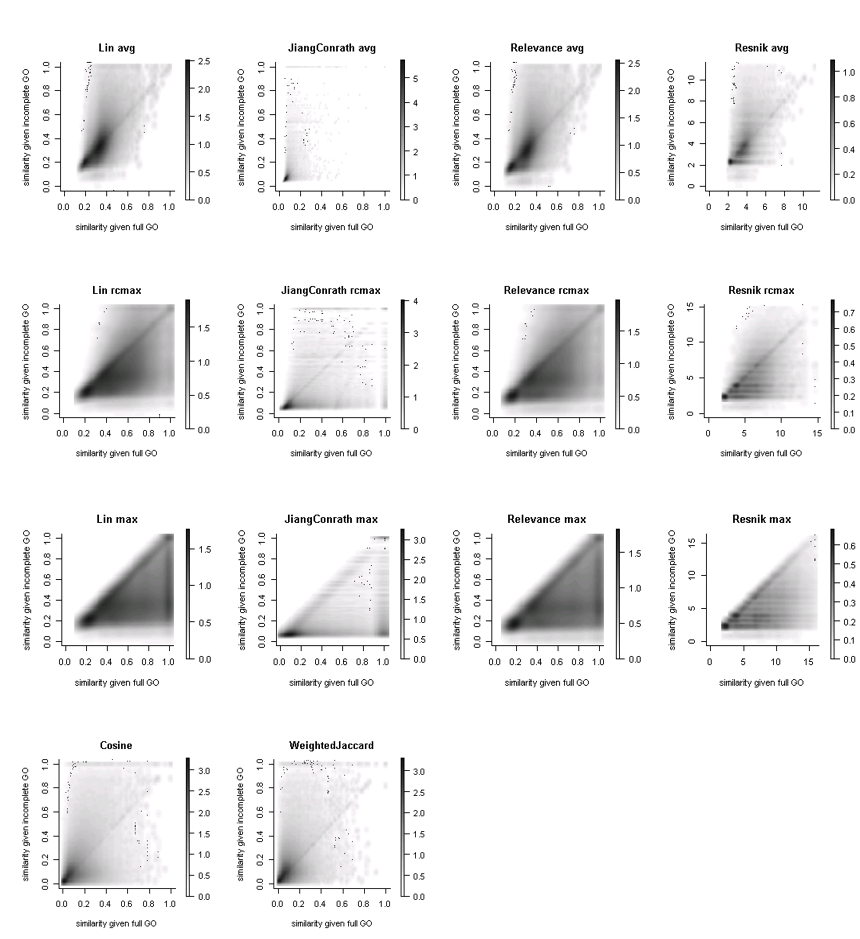


**Figure S2. Pairwise gene semantic similarities for complete vs. incomplete GO multicellular organism level process annotations.** Each point represents a unique gene pair with the value on X axis as their similarity for the complete annotations and the value on Y axis as their similarity for a random simulated incomplete set of annotations. Therefore, each gene pair is repeated 100 times in each plot, with each pair having the same similarity for complete annotations but a different similarity under a different simulated incomplete annotation set.


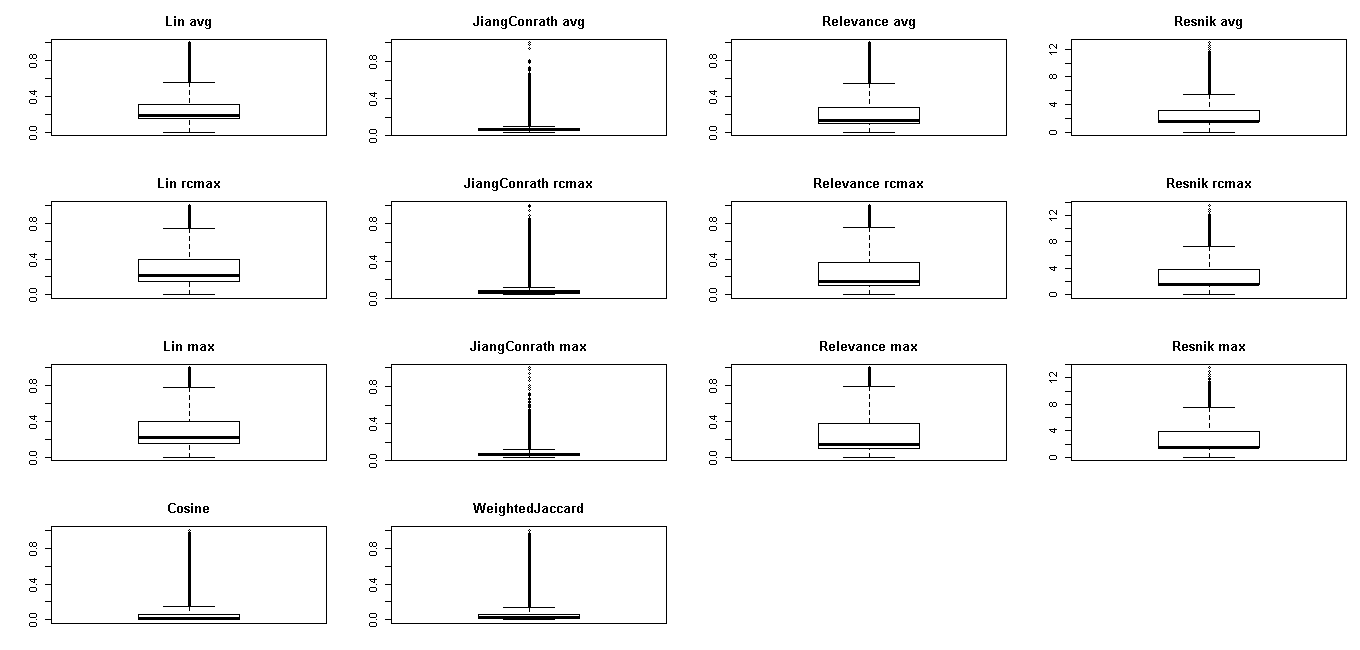


**Figure S3. Distributions of pairwise gene similarity scores for incomplete datasets.** Most similarity measures cluster in a narrow range that is greater than 0, resulting in a high density of scores in this range.


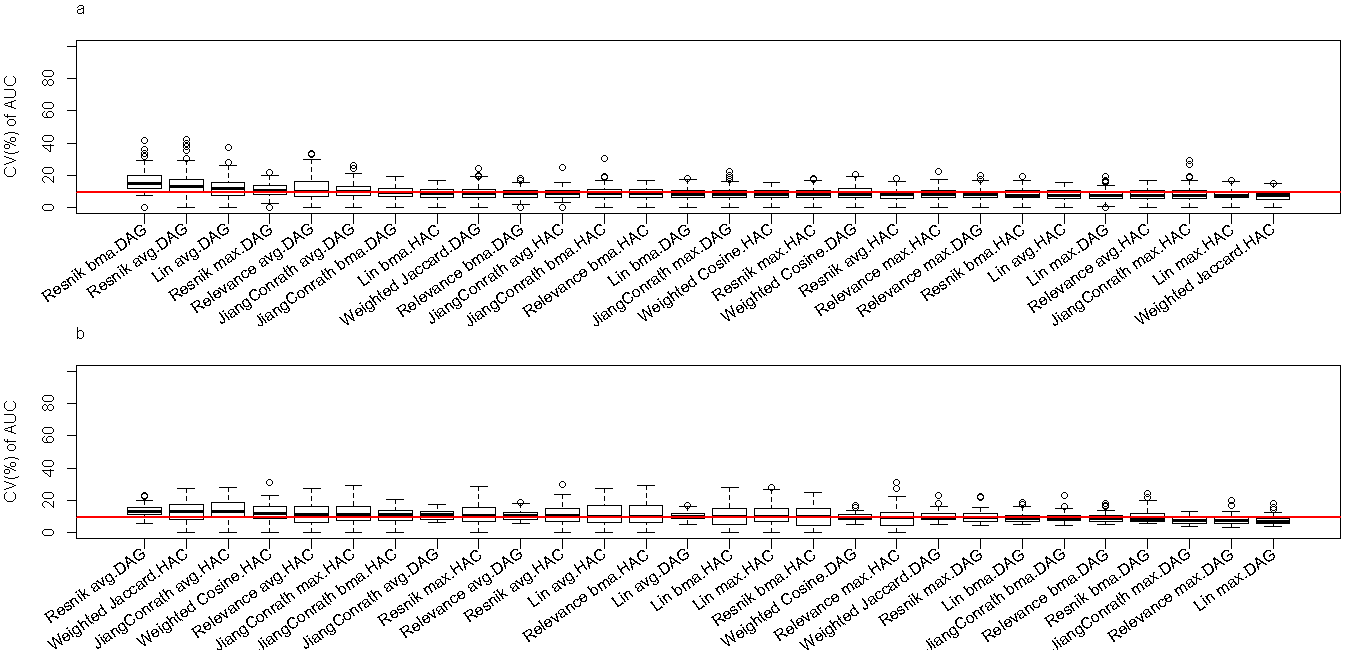


**Figure S4. Distributions of coefficient of variation (CV) (%) for clustering accuracy** **over 100 simulated incomplete annotation sets (see Figure 5).** (a) shows distribution for simulated cellular process annotation sets, and (b) shows distribution for simulated multicellular organism process annotation sets. CV is generally low, showing consistency of results between different simulated incomplete sets.
